# Chiral Separation of Mandelic Acid Derivatives Using Various Permethylated Cyclodextrin Selectors Containing Stationary Phases in GC

**DOI:** 10.3390/molecules30030451

**Published:** 2025-01-21

**Authors:** Zoltan Juvancz, Rita Bodáne-Kendrovics, Csaba Ágoston, Dóra Maklári, Csanad Csaba Voller, Zoltan Kaleta

**Affiliations:** 1 Rejtő Sándor Faculty of Light Industry and Environmental Engineering, Institute of Environmental Engineering and Natural Science, Óbuda University, Doberdó út 6, H-1034 Budapest, Hungary; bodane.rita@uni-obuda.hu (R.B.-K.); agoston.csaba@uni-obuda.hu (C.Á.); h8d7pw@tr.pte.hu (D.M.); 2 Institute of Bioanalysis, Medical School, University of Pécs, Honvéd Street 1, H-7624 Pécs, Hungary; 3 Department of Organic Chemistry, Semmelweis University, Hőgyes Endre Street 7, H-1092 Budapest, Hungary; voller.csanad@stud.semmelweis.hu; 4 Center for Pharmacology and Drug Research & Development, Semmelweis University, H-1085 Budapest, Hungary

**Keywords:** chiral separations, permethylated cyclodextrins, mandelic acid-based compounds, structure–chiral selectivity relationships

## Abstract

A good chiral separation usually results in a trial-and-error process; however, through systematic studies, certain principles can be established to correlate structure with chiral selectivity. These principles can then be applied to other chiral separations, reducing the time of developing chiral selective analytical methods. Using the model compounds, the established principles can be applied to a wider range of compounds. In this study, mandelic acid and its substituted derivatives were selected as model compounds to establish transferable rules. The chiral selectivity of 13 compounds was measured on various permethylated cyclodextrin selectors. Comparing the chiral selectivity of permethylated cyclodextrins with different ring sizes (α, β, and γ) provides further insight into the role of inclusion in the chiral selectivity of the cyclodextrin-based stationary phases. Different derivatives of acidic and hydroxyl functions of mandelic acids were tested. Ring- and alkyl-substituted mandelic acid enantiomeric pairs were also tested. By using these compounds, the role of hydrogen donor–acceptor interactions and dipole–dipole interactions and inclusions in chiral recognition processes were investigated. The chiral selectivity values were measured and extrapolated to the same temperature, for the sake of the comparison. Several general tendencies were concluded, which can be used for chiral separation of other enantiomer pairs.

## 1. Introduction

Chiral compounds are molecules defined by their inability to be superimposed on their mirror images, much like how our left and right hands are mirror opposites but cannot align perfectly when overlaid. This property arises from the specific three-dimensional arrangement of the center atom and its different ligands within the molecule, creating asymmetry and resulting in two distinct forms, known as enantiomers. This spatial configuration makes chiral compounds particularly significant in pharmacology, as the enantiomers of chiral drugs often exhibit vastly different effects on biological systems [1] and may also present a difference in therapeutic efficacy. This variation stems from the inherently chiral nature of biological molecules, such as enzymes and receptors, which interact with drugs in a highly specific, three-dimensional manner. The unique spatial structure of each enantiomer determines its interactions with biological targets, influencing both efficacy (e.g., ibuprofen [2]) and safety (e.g., thalidomide [3]). Consequently, separating enantiomers and accurately determining their ratios is of critical importance, particularly in the pharmaceutical industry [4].

Separating enantiomers involves utilizing the differences in their three-dimensional structure. Chiral recognition depends on precise geometric and chemical compatibility between interacting groups. Enantiomers form temporary complexes with chiral selectors, which differ in energetic stability. This process is often explained by the three-point interaction model [5]. According to this model, one enantiomer interacts with three points on the chiral selector, while the other interacts with only two. The additional interaction results in a more stable complex for one enantiomer, causing differences in retention times during chromatographic separations. Techniques such as gas chromatography (GC) [6], high-performance liquid chromatography (HPLC) [7], and capillary electrophoresis (CE) [8] exploit these differences for effective enantiomer separation.

Among the various chiral selectors, cyclodextrins (CDs) are particularly significant and widely utilized in capillary column techniques [9,10,11,12]. CDs are cyclic oligosaccharides composed of six, seven, or eight D(+)-glucopyranose units, corresponding to α-, β-, and γ-cyclodextrins, respectively [11]. These molecules can be chemically modified by substituting their hydroxyl groups with various neutral or ionic functional groups, enhancing their chiral recognition capabilities and making them versatile for a broad range of applications.

An essential feature of CDs is their inclusion properties [11]. They can encapsulate appropriately sized guest molecules within their cavities, facilitating chiral recognition. CDs exhibit vast chiral recognition capabilities due to several factors [9,12]:

- **Numerous Chiral Centers**: The asymmetric carbon atoms (e.g., 35 in β-CD) of glucose units differ from each other, each with unique configurations. Their twisted shapes create diverse functional group arrangements, enabling broad recognition spectra compared to linear molecules like amylose.
- **Functional Group Modifications**: Derivatized CDs possess (e.g., phosphate, sulfate, amino derivatives) enhanced interaction capabilities and selectivity.
- **Structural Flexibility**: CDs may alter their shape in order to adapt to the functional groups of the analyte, thus increasing chiral selectivity.
- **Ionization States**: Ionizable CDs exhibit variable selectivity depending on their ionization states.
- **Solvent Interaction**: Both analyte and solvent molecules may enter the cavities of CDs, depending on the mobile phase and buffer composition.

CDs typically favor interactions with functional groups at the alpha position near the asymmetric center, though it is possible for them to interact with groups at beta, gamma, or other positions. CDs have a multimodal nature that allows multiple recognition mechanisms to occur for various analytes. Small structural changes in analytes may result in different interaction mechanisms, including elution reversal. Though inclusion within the CD cavity is often a key interaction, it may not always be necessary for chiral recognition.

Cyclodextrins dominate as chiral selectors in GC and CE due to their compatibility with capillary columns [9,12]. Although their moderate chiral selectivity results from structural flexibility, the high performance of these techniques compensates for this limitation. Predicting chiral recognition parameters for specific enantiomer pairs remains challenging, often necessitating trial-and-error approaches in separation development.

Systematic studies using model compounds, such as mandelic acid [13], have provided valuable insights into CD selectivity mechanisms. Mandelic acid, containing an alcoholic and an acidic function with an aromatic ring, serves as an excellent model due to its potential for functional modifications [14]. These modifications enable further exploration of chiral interactions, aiding the development of separation methods for other enantiomeric pairs [15].

This research builds on our previous studies, where the chiral selectivity of various mandelic acid derivatives was compared on a single stationary phase [13]. Here, we observe the selectivity of mandelic acid derivatives across multiple chiral stationary phases, similar to our work with other model compounds [16,17]. Our objectives include identifying optimal analytical conditions and exploring the elution orders of optical isomers. Determining the elution order of minor enantiomers is particularly crucial for trace impurity analysis [18].

## 2. Results

The mandelic acid could not be analyzed in gas chromatographic mode without derivatization, because it has a high boiling point (321.8 °C) and a very polar character. Various analytical derivatives of mandelic acid and substituted mandelic acids were synthesized to get appropriate derivates for gas chromatographic analysis. The synthesized derivatives offered appropriate formats for analyses. Selectivity differences of tested compounds also gave a deeper insight into the chiral recognition features of the selectors.

Natural logarithmic values of chiral selectivity of every separated enantiomeric pair showed linearity in a function of the reverse value of the absolute temperature of analysis (lnα—1/T). The corrected retention times (t_R_,) produced similar linear characters (lnt_R_—1/T) to chiral selectivity. The linearity of the lnα—1/T relationship allowed the calculation of chiral selectivity, retention times, and Kovats retention index (KI) values [19] at 100 °C with linear extrapolation. The calculated boiling points [20] of derivatives were also taken into consideration, which were useful to determine the intensity of inclusion phenomena. Namely, the relatively increased KI values compared with their boiling points showed the inclusion of the tested derivatives. We also determined the retention orders of enantiomers, if we had non-racemic compounds. In this way, the minor first elution orders can be evaluated with the appropriate combination of mandelic acid derivatives and selectors for any mandelic acid product even in cases of trace-level enantiomeric impurity [18].

The linearity of the lnα—1/T relationship also proved that the given separation belonged to only one chiral recognition mechanism [21,22].

The results were divided into three groups for detailed discussions:

- Various analytical derivatives, where mandelic acid was the starting material;
- Various alkyl chain-substituted derivatives of mandelic acid were the starting material;
- Various ring-substituted derivatives of mandelic acid were the starting material.

Different analytical derivatives of these materials were tested at least three various temperatures.

### 2.1. Results with Analytical Derivatives, Where Mandelic Acid Was the Starting Materials

**Figure 1:**
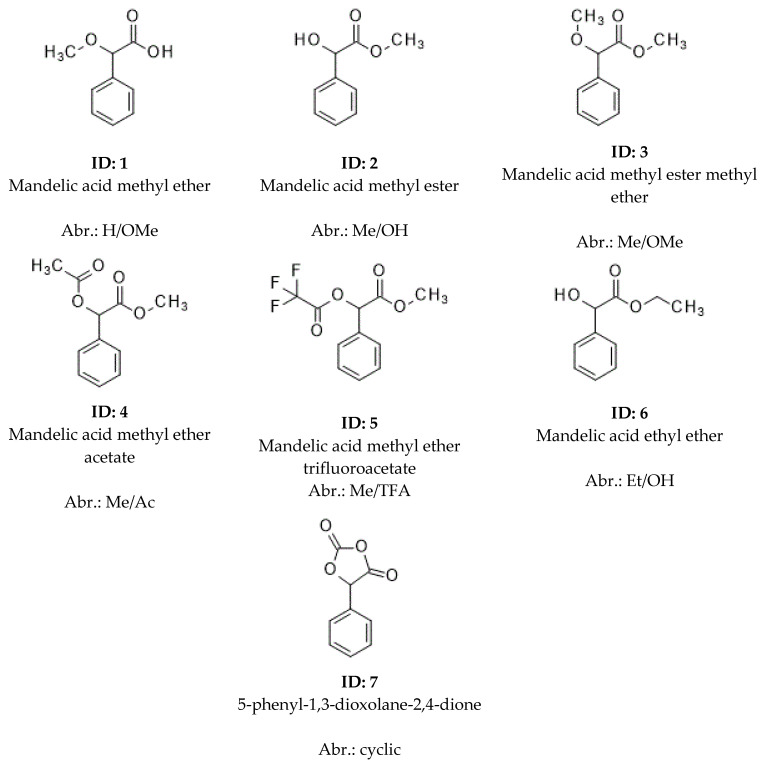
The structures of tested analytical derivatives of mandelic acid are shown, where mandelic acid was the starting material. This figure contains structural formulas, identification numbers (bold), names derived from mandelic acid, and abbreviations of the compounds. The system of abbreviations is as follows: the first symbols refer to the states of carboxyl groups, while the second refers to the states of hydroxyl groups in the abbreviations. The symbols of acid groups are as follows: H, underivatized acid; Me, methyl esters; Et, ethyl ester. The abbreviations of hydroxyl groups are as follows: OH, underivatized hydroxyl; Ac, acetate; TFA, trifluoro acetate; OMe, methyl ether. The 1,3-dioxolane-2,4 dione cyclic derivative of carboxyl and alcoholic groups are signed: cyclic.

The structures of tested analytical derivatives of mandelic acid (1–7) are shown in Figure 1.

The achieved results with various analytical derivatives, where mandelic acid was the starting material, are summarized in Table 1. The data of Table 1 contain not only the identification numbers (ID.), states of the functional groups, and abbreviations of derivatives, but also their calculated chiral selectivity values, Kovats retention index values [19], elution orders of isomers on various selectors, and their calculated boiling point [20]. The letters under the elution order show the first eluting enantiomers. Of course, the different selectors are also signed in Table 1.

The various stationary phases that were applied showed different retention times for the tested compounds at the same temperature, even for the normal alkanes. This fact indicated that they have different achiral retentions; therefore, we used the Kovats retention index (KI) values of tested compounds instead of their retention time. In this way, the relative retention differences became more emphasized, making it possible to observe the inclusion phenomena of the different tested compounds. In those cases, where any chiral separation was not observed, a value of <1.01 was written in the selectivity cells, and nothing was written in the elution order cells. The calculated boiling points of derivatives were also attached, which were useful to recognize the intensity of inclusion phenomena.

For the sake of better identification of the discussed derivatives, their serial numbers and the abbreviations in Table 1 are added in brackets in the text.

Any of the derivatives of Table 1 were somehow chirally separated by at least two selectors. Even a 1.107 selectivity value has been established using α-cyclodextrin-containing selector (α-CD) for the cyclic (7) derivative. The permethylated β-cyclodextrin (β-CD) proved to be the best chiral selector due to the fact that it separated all isomers and gave the second-highest selectivity value 1.085 in H/OMe (1) form.

The alcoholic OH groups of the derivatives (2, 6) offered good chiral recognition abilities in every instance. The highest selectivity was on β-CD selector 1.054 for Et/OH (6) and 1045 for Me/OH (2) from the underivatized alcoholic derivatives. It is possible that the ethyl ester could immerse more deeply into the cavities of β-CD selectors than the methyl ester.

The α-CD was also a good selector for Me/OH (2) and Et/OH (6) with selectivity values of 1.036 and 1.035, respectively. The permethylated γ-CD (γ-CD) showed the smallest selectivity toward these derivatives, 1.029 for Me/OH (2) and 1.025 for Et/OH (6). It is interesting to note that the ethyl ester Et/OH (6) derivatives produced similar chiral selectivity values to the methyl ester Me/OH (2) derivatives, in spite of the general practice [21]. In these cases, the H-bond donor ability played a key role in the chiral recognition on every tested stationary phase, according to the general observations [21,22].

**Figure 2:**
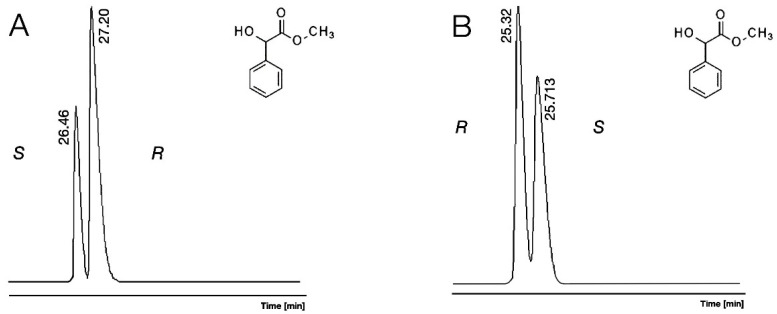
The elution reversal of enantiomers of mandelic acid methyl ester (Me/OH, 2) with *R* isomer excess on permethylated α-cyclodextrin (α-CD) showed on subfigure (**A**) and permethylated γ-cyclodextrin (γ-CD) showed on subfigure (**B**) containing chiral selectors.

The underivatized acid functions of acid (H/OMe, 1) also offered excellent chiral selectivity on β-CD (1.085) and γ-CD (1.053). The stronger H-bond donor features of the carboxyl group of mandelic acid (H/OMe, 1) might be the reason for their improved chiral selectivity value of 1.085 on β-CD, which is the second best-achieved value amongst the data of Table 1. The 1.053 selectivity value of H/OMe (1) was also higher on the γ-CD selector than what was measured for Me/OH (2). Probably the free carboxylic function could make intensive interactions with the H-bond acceptor oxygen atoms of the selectors in their upper rims in these cases. On the other hand, the α-CD did not produce any chiral selectivity toward acidic H/OMe (1). Thus, our understanding is that the α-CD selector had a different mechanism than β-CD and γ-CD selectors, which resulted in no chiral resolution toward H/OMe (1). The different chiral recognition mechanisms of these selectors became obvious from their different elution order data. The Me/OH (2) and Et/OH (6) derivatives show *R* before *S* elution orders on β-CD and γ-CD selectors, but *S* before *R* elution orders were observed for these derivatives on the α-CD selector. The different elution orders of Me/OH (2) on α-CD and γ-CD are illustrated in Figure 2.

Conditions: instrument, Shimadzu 17A/QP5000 GC/MS,

**A**: Conditions: column, 25 m × 0.25 mm; stationary phase, Alfa Dex 120; carrier, Helium (50 cm/s); temperature, 110 °C.

**B**: Conditions: column, 25 m × 0.25 mm; stationary phase, Gamma Dex 120; carrier, Helium (50 cm/s); temperature, 90 °C.

The role of the H-bond donor ability became more emphasized in the chiral recognition mechanisms comparing the chiral selectivity values of mandelic acid methyl ether methyl ester (Me/OMe, 3) to derivatives that had H-bond abilities (1, 2, 6). No chiral selectivity or decreased selectivity was measured for mandelic acid methyl ether methyl ester (Me/OMe, 3) that lacked H-bond donor ability. For example, the Me/OH (2) showed a 1.029 chiral selectivity value, but no selectivity was observed for Me/OMe (3) on the γ-CD selector at 100 °C.

**Figure 3:**
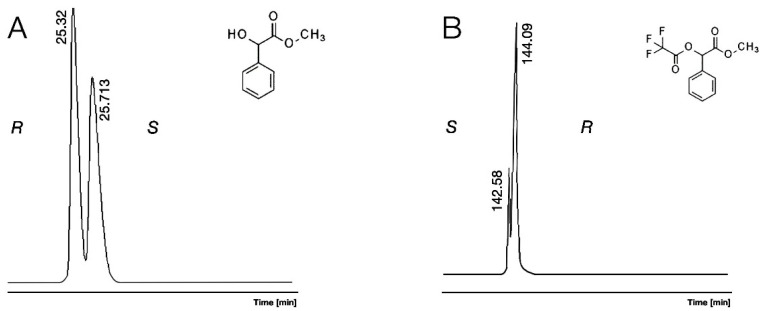
The elution reversal of enantiomers of mandelic acid methyl ester (Me/OH, 2) showed on subfigure (**A**) and mandelic acid methyl ester trifluoro acetate (Me/TFA, 5) showed on subfigure (**B**) with *R* isomer excess on permethylated γ-cyclodextrin (γ-CD)-containing chiral selectors.

The high selectivity caused by H-bond donor abilities of free hydroxyl functions of Me/OH (2) and Et/OH (5) also decreased with acetylation or trifluoro-acetylation of their hydroxyl functions. The acylated derivatives of mandelic acid methyl ester (Me/Ac, 4) and mandelic acid methyl ester trifluoro acetate (Me/TFA, 5) did not have H-bond donor characters, which explains their decreased chiral selectivity, which is illustrated in Figure 3. However, their improved H-bond acceptor ability produced some chiral selectivity in the cases with acylated methyl esters derivatives (Me/Ac, 4) on every selector

The measured retention orders of various derivatives were the same, *S* before *R* in the case of α-CD. On the other hand, the observed elution order *R* before *S* of Me/OH (2) reversed to *S* before *R* in the cases of Me/Ac (4), Me/TFA (5), and Cyclic (9) on the β-CD selector and on the γ-CD selector with the exception of the Cyclic (9) derivative. This retention reversal is illustrated in Figure 3.

Conditions: instrument, Shimadzu 17A/QP5000 GC/MS; column, 25 m × 0.25 mm; stationary phase, Gamma Dex 120; carrier, Helium (50 cm/s); **A**: mandelic acid methyl ester (Me/OH, 2), 110 °C. **B**: mandelic acid methyl ester trifluoro acetate (5), 90 °C.

**Figure 4:**
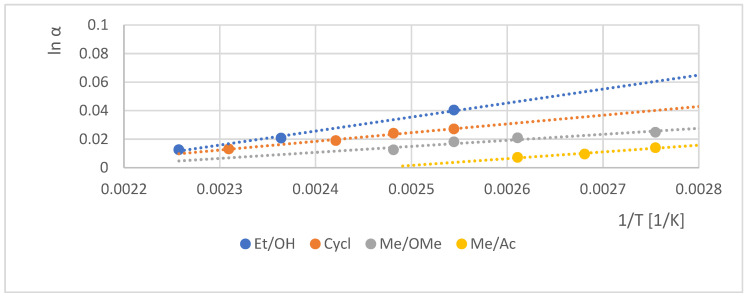
Chiral selectivity (ln α) as a function of the reverse value of analyses temperatures (1/T) of various mandelic acid derivatives on permethylated β-cyclodextrin-containing chiral selector under GC conditions. Symbols: •: Et/OH (2); •: Cyclic (7); •: Me/OMe (3); •: Me/Ac (4). Conditions: instrument, Shimadzu 17A/QP5000 GC/MS; column, 25 m × 0.22 mm; stationary phase, Cydex B; carrier, Helium (50 cm/s); temperature range, 80–170 °C.

The role of the H-bond ability was strengthened with the comparison of the chiral selectivity (lnα) dependence of various derivatives on the β-CD selector in a function of temperature (1/T), which is demonstrated in Figure 4.

The high temperature dependency of chiral selectivity further supported the key role of the hydrogen donor ability in the chiral recognitions in the case Et/OH (6) compared to other derivatives shown in Figure 4.

Despite the lack of H-bond donor ability, the 1,3-dioxolane-2,4 dione derivative (cyclic, 7) showed a rather high selectivity value on the α-CD and β- CD selectors. The highest selectivity value of 1.103 was measured with this derivate using the α-CD selector. The extended ring system of cyclic (7) gave a rigid structure, which fit well into the cavity of the α-CD. The five-member 3-dioxolane-2,4 dione ring system could deeply immerse guests into the cavity of α-CD [10]. On the other hand, the cyclic (7) created only a loose and weak inclusion complex with the γ-CD selector, which could not compensate for the lack of H-bond donor groups, resulting in unrecognized chiral selectivity.

Generally, the β-CD selector offered higher chiral selectivities toward the various derivatives of mandelic acids than α-CD and γ-CD except for Me/Ac (4) and Me TFA (5) derivatives. In the case of H/MeO (1), however, the γ-CD selector was the most selective and showed a protruding KI value. The α-CD showed regularly higher selectivity than γ-CD toward various derivatives of mandelic acid. It seems that the structures of mandelic acid derivatives fit better into the chiral recognition features of β-CD than the α-CD and γ-CD. On the other hand, the γ-CD showed the highest KI values for various derivatives of mandelic acids, which was followed by the KI values on β-CD. Probably the γ-CD created the strongest inclusion complexes with the mandelic acid derivatives. These facts suggest that the chiral recognitions of these selectors did not correlate strongly with the values of the inclusion forces in several cases. On the other hand, the inclusions became more important in the cases of ring-substituted mandelic acids than in the cases of mandelic acid derivatives.

A loose correlation could be established between the chiral selectivity values of the derivatives and their improved KI values while also taking their boiling points into consideration. However, some inclusions were necessary for the chiral recognition.

The minor first elution order is advantageous in the analysis of highly enantiomer pure product [18]. Therefore, the elution orders of the isomers were tested. Elution order reversals were tested during our experiments. The α-CD selector produced *S* before *R* elution orders in every case (Figure 2A). The β-CD and γ-CD selectors showed an *R* before *S* elution order with derivatives having underivatized hydroxyl groups (1, 2, 6, Figure 2B). On the other hand, these selectors produced an *S* before *R* elution order for the acetate (Me/Ac, 4, Figure 3B), TFA (Me/TFA, 5), and 1,3-dioxolane-2,4 dione (cycl, 7) derivatives. Those derivatives, which have H-bond acceptor features, can be the reason for the different recognition mechanisms than derivatives that do not have H-bond donor characters. The methyl ester methyl ether derivatives (Me/OMe, 3) do not have a strong H-acceptor character; therefore, their retention orders were the same as H-bond donor derivatives. Similar retention reversals of the mandelic acid methyl ester acetate derivatives (*R* before *S*) were already observed on the 2,3-Di-O-acetyl-6-O-tert-butyldimethylsilyl-gamma-cyclodextrin selector [13].

### 2.2. Results with Analytical Derivatives, Where Alkyl Chain-Substituted Mandelic Acids Were the Starting Materials

**Figure 5:**
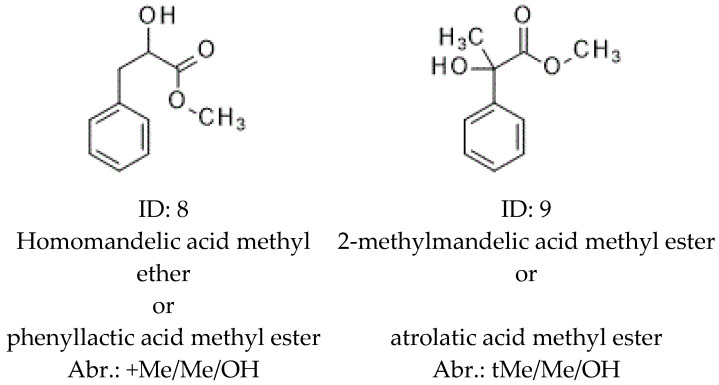
The names, structures, identification numbers, and abbreviations of tested analytical derivatives, where alkyl-substituted mandelic acids were analyzed. The system of the abbreviations is the same as in Figure 1, with the addition of positions of the methyl substituents in the prefix.

Two alkyl chain-substituted mandelic acids were tested also on α-CD, β-CD, and γ-CD selectors (Figure 5). One of them was phenyllactic acid methyl ester (homomandelic acid methyl ether, ID 8, +MeMeOH), where a methyl group was inserted between the chiral carbon atom and the carbon atom of the carboxyl group of the mandelic acid. The other was the atrolatic acid methyl ester (2-methylmandelic acid methyl ester, ID: 9, tMeMeOH), where a methyl group was attached to the asymmetric carbon atom of the mandelic acid.

The results of the alkyl chain-substituted mandelic acid derivatives are summarized in Table 2.

Table 2 is similar to Table 1 in its structure and its abbreviations. Unfortunately, we only had racemic mixtures of the alkyl chain-substituted derivatives; therefore, Table 2 only contains methyl ester derivatives and lacks elution orders. Table 2 also contains the data of mandelic acid methyl ester (2. Me/OH), for the purpose of better comparison of these tendencies.

The phenyllactic acid methyl ester (+Me/Me/OH, 8) and atrolactic acid methyl ester (9, tMe/Me/OH) produced rather different chiral selectivity profiles despite, both of them being substituted with one carbon atom unit compared to Me/MeOH (2). The +Me MeOH (8) almost lost its chiral selectivity on the tested selectors. Only β-CD showed some (1.015) chiral recognition properties. The rigid phenyl ring was situated further, with one methylene unit from the chiral center. Probably this distance gave more flexibility around the chiral center, causing decreased chiral recognition abilities. On the other hand, the tMeMeOH (9) produced excellent selectivity values. The tMeMeOH (9) produced bigger selectivity values than Me/MeOH (2) on α-CD and β-CD selectors. It also yielded an acceptable selectivity value on the γ-CD selector. The tertiary carbon resulted in a more rigid system than the secondary carbon of Me/Me/OH (2), which offered better selectivity chiral recognition abilities for tMeMeOH (9) on selectors having smaller ring sizes.

The KI values of tMe/Me/OH (9) are smaller than the values of Me/OH (2) on every selector in spite of the boiling points of these derivatives, which were rather similar to each other. This phenomenon might be caused by the floppier inclusions of tMe/Me/OH (9). Lower KI values of tMe/Me/OH (9) were experienced. These can be caused by the lower acidity of the tertiary hydroxy group’s weaker H-bond donor character. The tertiary hydroxy group of tMe/Me/OH (9) was also partly shielded by phenyl and ester groups.

The modification of the α-carbon had a significant effect on the chiral selectivity of the tested molecules. The chiral selectivity of the more flexible +Me/Me/OH (8) resulted in a lower value than that of the more rigid tMe/Me/OH (9).

### 2.3. Results Where Various Ring-Substituted Derivatives of Mandelic Acid Were the Staring Materials

**Figure 6:**
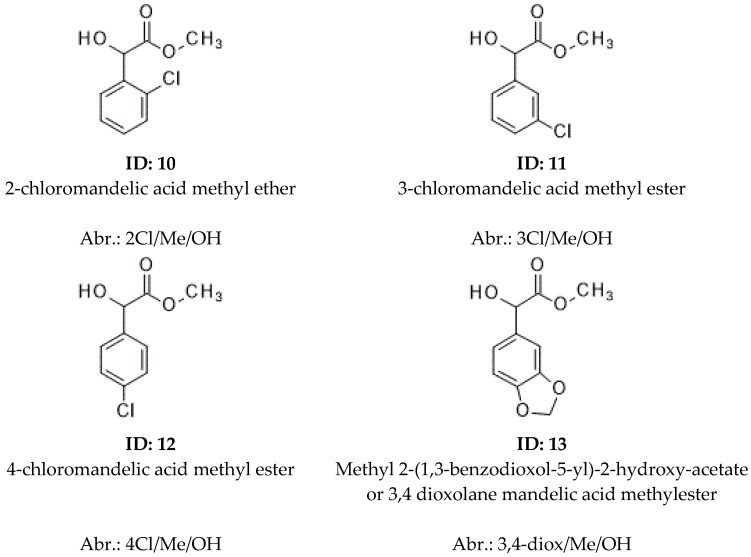
The structure, identification numbers, and abbreviations of the tested ring-substituted mandelic acid. The abbreviation system is similar to Figure 5. The signs of the ring substitution are the following prefixes: 2,3 diox/, 3,4 dioxolane; 2Cl/, ortho; 3Cl/, meta, and 4Cl/para, according to the positions of their substitutions.

The isomers of a bi-substituted phenyl ring (ortho, meta, para) can give rather different inclusion properties [11]; therefore, the chlorine ring-substituted and 3,4 dioxolane isomers of mandelic acid were also tested (Figure 6).

The ring substitution significantly influenced the inclusion properties of tested compounds. The results of the ring-substituted mandelic acids are summarized in Table 3.

The structure of Table 3 logically follows the previous tables. Unfortunately, we had only racemates from 3,4-dioxolane (13); therefore, the elution order of its enantiomers is missing from Table 3.

The position of the ring substitutions of mandelic acid influenced a lot of the chiral selectivity of various analytes. No chiral selectivity was recognized for ortho 2Cl/Me/OH (10) on α-CD and β-CD selectors, but a 1.027 value was achieved for meta 3Cl/Me/OH (11) on the α-CD and 1141 on β-CD. Probably the ortho chlorine substitutions made this molecule so wide that it could not immerse into the cavity of the α-CD and β-CD. On the other hand, the broadest-sized cavity of the γ-CD selector is appropriate for the inclusions of the 2Cl/Me/OH (10) producing 1.029, a good selectivity value. It is interesting to note that the KI values of 2Cl/Me/OH (10) were lower on α-CD and β-CD than on the γ-CD selector. These facts suggest the lack of inclusions on two smaller-sized selectors. It seems that the chiral selectivity of α-CD had a close correlation with its inclusion properties, because the 3Cl/Me/OH (11) had the highest chiral selectivity as well as KI value from the chlorine-substituted isomers.

On the other hand, the selectivity of β-CD did not correlate with its inclusion properties. The inclusion properties were estimated from the KI values of the chlorine isomers. The highest selectivity was 1.170 for 4Cl/Me/OH (12) on β-CD, but meta substitution produced the lowest KI values among the chlorine isomers in this selector in spite of 3Cl/Me/MeOH providing a rather high (1.141) chiral selectivity.

**Figure 7:**
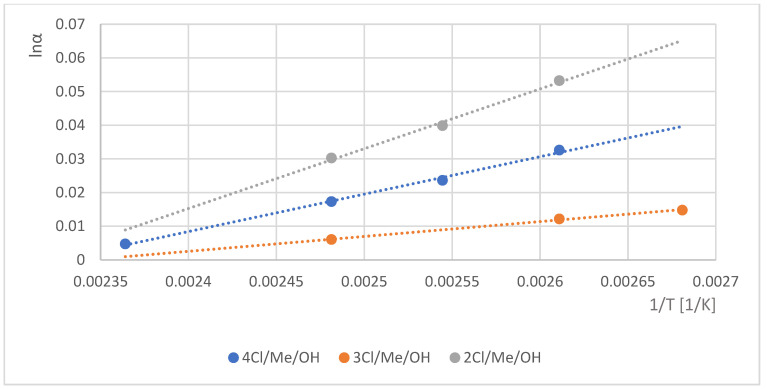
The natural logarithmic values of chiral selectivity (lnα) in the function of reverse absolute temperature (1/T). Symbols: •: 2Cl/Me/OH, (10); •: 3Cl/Me/OH (11); •: 4Cl/Me/OH (12) Conditions: instrument, Shimadzu 17A/QP5000 GC/MS; column, 25 m × 0.25 mm; stationary phase, Gamma Dex 120; carrier, Helium (50 cm/s); temperature range, 100–150 °C.

It is possible that the shielding effects of chlorine substituents on the meta position of 3Cl/Me/MeOH (11) and more pronounced extent on the ortho position of 2/Cl/Me/OH (10) could decrease the H-bond donor abilities of the hydroxyl groups. The high H-bond donor ability of the isomers was influenced by the temperature dependency of their chiral selectivity values on the γ-CD selector (Figure 7).

**Figure 8:**
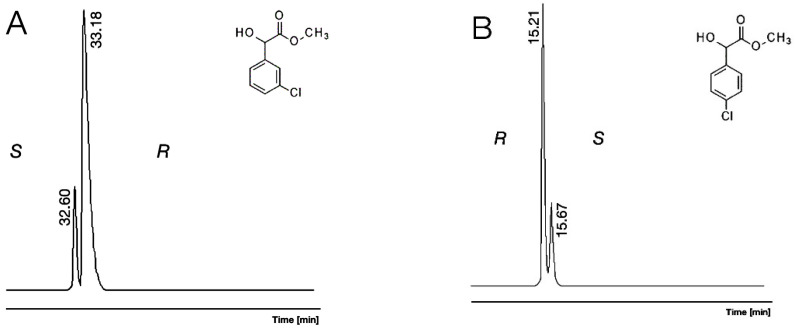
The elution reversal of enantiomers of 3-chloro mandelic acid methyl ester (3Cl/Me/OH 11) showed on subfigure (**A**) and 4-chloro mandelic acid methyl ester (4Cl/Me/OH, 12) showed on subfigure (**B**), with *R* isomer excess on permethylated β-cyclodextrin (β-CD)-containing chiral selectors.

It seems the chiral selectivity of γ-CD had a loose correlation with its inclusion feature. The chiral selectivity of γ-CD increased in the order of meta, ortho, and para (10, 11, 12 respectively) which had the same tendencies as their KI values for chlorine-substituted isomers. It is interesting to note that the elution order reversal was from *S* to *R* between the 3Cl/Me/OH (11) and 4Cl/Me/OH (12) on β-CD selectors; this is illustrated in Figure 8.

Conditions: instrument, Shimadzu 17A/QP5000 GC/MS; column, 25 m × 0.25 mm; stationary phase, Cydex-B; carrier, Helium (50 cm/s)

**A**: 3-chloro mandelic acid methyl ester (3Cl/Me/OH, 11) and mandelic acid methyl ester (Me/OH, 2), 130 °C.

**B**: 4-chloro mandelic acid methyl ester (4Cl/Me/OH, 12), 150 °C.

The cyclic 3,4-diox/Me/OH (13) gave good chiral selectivity on α-CD and β-CD selectors, but it was very small on the γ-CD-containing stationary phase. Probably the five-membered ring system of 3,4-diox/Me/OH (13) fitted well to the smaller cavity-sized α-CD and β-CD selectors.

## 3. Materials and Methods

### 3.1. Materials

The mandelic acid and variously substituted mandelic acids (2-chloromandelicacid, 3-chloromandelic acid, 4-chloromandelic acid, and 3,4-methylenedioxymandelic acid) were purchased from Merck LifeScience Kft, Budapest, Hungary, and phenyllactic, mandelic acid methyl ether and an atrolactic acid sample were generously donated by Prof. Karol Kacprzak (the Department of Chemistry, Adam Mickiewicz University, Poznan, Poland). Methanol, ethanol, ethyl acetate, chloroform, anhydrous, Na_2_SO_4_, NaHCO_3_, HCl, H_2_SO_4_, acetyl chloride, trifluoroacetic anhydride, triethyl amine, and triphosgene were products of Merck LifeScience Kft, Budapest, Hungary. N-alkanes were products of Reanal Ltd. (Budapest, Hungary). All of the applied chemicals were purissimum grade.

### 3.2. Instrumentation

The GC-MS experiments were performed with a Shimadzu 17A/QP5000 (Simkom Kft., Budapest, Hungary).

on-line coupled gas chromatographic/mass spectrometric instrument. The following chiral stationary phases were used: Alpha Dex (30 m × 0.25 mm, d_f_ 0.25 μm) mixture of silicone polymer and permethyl α-CD (Merck LifeScience Kft, Budapest, Hungary); Cydex-B column (25 m × 0.22 mm, d_f_ 0.25 μm) mixture of silicone polymer and permethyl β-CD, (SGE, Melbourne, Australia); Gamma Dex (30 m × 0.25 mm, d_f_ 0.25 μm) mixture of silicone polymer and permethyl γ-CD (Merck LifeScience Kft, Budapest, Hungary). Helium was applied as a carrier gas. A temperature-controlled drying cabinet was used for heat treatments during the derivatization processes. Reacti-Vials were purchased from Merck LifeScience Kft, Budapest, Hungary.

### 3.3. Experimental Procedures

The low volatility and high polarity of mandelic acid made it necessary to derivatize one of its functional groups (or both) for its gas chromatographic analyses.

Preparing alkyl ester derivatives of acidic groups was carried out according to standard procedures [23]. In the cases when both isomers and racemic mixtures were available, 0.066 mmol racemic compounds and 0.033 mmol (*R*) isomer were mixed together in a Reacti-Vial and dissolved in 1 mL of methanol or ethanol. In this way, the determination of the retention order of *R* and *S* isomers was possible. One drop of concentrated HCl or H_2_SO_4_ was added as a catalyst. When we had only racemic mixtures, 0.1 mmol samples were taken. The closed vials were heated at 80 °C for 2 h. After the reaction mixtures were cooled down, they were quenched with 0.5 mL of distilled H_2_O, and 2 mL of ethyl acetate was added. The aqueous phases were neutralized with a 0.1 mol/L NaHCO_3_ solution. The phases were separated using syringes. The organic phases were dried with anhydrous Na_2_SO_4_. The drained solutions were dried under N_2_ streams. The dried materials were dissolved in 3 mL ethyl acetate, and 1 mL of each was analyzed in the GC/MS instrument.

Preparing acetate derivatives of alcoholic groups was carried out according to standard procedures [23]. A total of 1 mL of the ethyl acetate solutions of the ester (0.033 mmol) was reacted with 90 μL (0.066 mmol) of acetyl chloride, which was added drop by drop into Reacti-Vials. The Reacti-Vial was closed and heated for 2 h at 80 °C. The reaction mixture was cooled down and quenched with 0.5 mL of distilled H_2_O, and 2 mL of ethyl acetate was added. The aqueous phase was neutralized with a 0.1 mol/L NaHCO_3_ solution. The phases were separated using syringes. The organic phase was dried with anhydrous Na_2_SO_4_, and the drained solution was dried under N_2_ streams. The dried material was dissolved in 1 mL ethyl acetate and analyzed via a GC/MS instrument. Preparing trifluoro acetate derivatives of alcoholic groups was carried out according to standard procedures [23]. A total of 1 mL of the ethyl acetate solutions of the ester (0.033 mmol) was reacted with 90 μL (0.066 mmol) of trifluoro acetic acid anhydride and 90 μL triethyl amine, which were added drop by drop into Reacti-Vials. The Reacti-Vial was closed and heated for 2 h at 80 °C. The reaction mixture was cooled down and quenched with 0.5 mL of distilled H_2_O, and 2 mL of ethyl acetate was added. The aqueous phase was neutralized with a 0.1 mol/L NaHCO_3_ solution. The phases were separated using syringes. The organic phase was dried with anhydrous Na_2_SO_4_, and the drained solution was dried under N_2_ streams. The dried material was dissolved in 1 mL ethyl acetate and analyzed via a GC/MS instrument.

5-phenyl-1,3-dioxolane-2,4-dione was synthesized according to a known procedure [24]: To a solution of α-hydroxy acid (10 mmol, dried in vacuum for 2 h) in dry THF was added activated charcoal (160 mg, activated at 120 °C for 2 h) under a N_2_ atmosphere. Then, triphosgene (6.7 mmol, 1.98 g) was added to this mixture. The resulting mixture was stirred at rt for 20 h. The organic solution was collected by filtration and concentrated in a vacuum. The crude product was washed with anhydrous PE twice and then recrystallized from given solvents three times to obtain a white crystal solid.

We tested the chiral selectivity and Kovats retention indices [19] of different derivatives of mandelic acid. Alkyl chain-modified, ring-substituted, and ring-expanded mandelic acid compounds were also derivatized and measured as mandelic acid.

### 3.4. Measurements

We tried to make appropriate chiral separations, at least three, at different analysis temperatures. The three measured points were enough to calculate lnα—1/T curves, where α is the measured chiral selectivity, and T is the absolute temperature (K) of the given separations. Our purpose was to gain α > 1.02 values in 5–120 min time intervals; however, it was not possible to achieve our goals on every occasion. In these cases, decreased analysis temperatures (longer retention times) were applied; otherwise, we could only achieve selectivity values lower than 1.02.

### 3.5. Calculations

For the sake of comparison, the measured and calculated chiral selectivity and Kovats retention index (KI) [19] values of different derivatives are given at 100 °C. The Kovats retention indices show the differences in the interaction energies of various derivatives better than their retention times. The chiral selectivity values and retention times were measured for every tested molecule at least three different temperatures. These data made it possible to count the lnα—1/T functional relationships and calculate the chiral selectivity values of tested compounds at 100 °C. The measured retention times (t_R_) of analytes also made it possible to count their KI values. If the enantiomers were separated, their mean retention times were the bases of KI calculations. The lnt_R_—1/T functional relationships were appropriate to calculate the retention times of analytes at 100 °C. The calculated and measured retention times of the tested derivatives were compared to the measured retention times of n-alkanes at 100 °C. The results of these comparisons gave the KI values of tested materials. We also calculated the boiling points [20] of different derivatives to gain information for the inclusion of the test molecules.

## 4. Conclusions

The H-bond donor ability of the hydroxy groups improved the chiral selectivity on every tested selector. However, this is not true in the case of H/OMe with underivatized acidic function, because it could increase the selectivity only on larger-sized β-CD and γ-CD selectors. Generally, the β-CD selector proved the most effective selectors for the tested compounds. It seems the inclusions were not the determining factor of high chiral selectivity values in several cases. However, some inclusion is necessary for chiral recognition, because the lack of inclusion prevents the chiral separations of larger ortho chlorine-substituted test materials on α-CD and β-CD selectors. On the other hand, of the five ring-containing systems, cyclic (7) and 3,4-diox (13) showed the highest selectivity values on α-CD selectors. Probably the five-membered structures fit well to the cavity size of α-CD. The loose inclusion interactions might not give extra retention but better directions for chiral recognition.

If one selector fails to separate a certain enantiomeric pair, a successful chiral separation of it can be achieved by changing the ring size of the cyclodextrin selectors or making an analytical derivatization. It is recommended to use α-CD selectors if the mandelic acid sample has high *R* enantiomer excess, but it is better to use β-CD or γ-CD selectors in the case of high *S* excess.

## Figures and Tables

**Table 1:** Chiral selectivity, Kovats retention indices (KIs), elution orders, and their calculated boiling point of mandelic acid derivatives, where mandelic acid was the starting material on different cyclodextrin-based selectors at 100 °C.

ID	Abr.	Acid ^a^	Alcohol ^b^	α-CD ^e^			β-CD ^f^			γ-CD ^g^			bp ^d^
				Selectivity ^c^	KI	Elution Order	Selectivity ^c^	KI	Elution Order	Selectivity ^c^	KI	Elution Order	
1	H/OMe	H	CH_3_	<1.01	1767		1.085	2114	*R*	1.053	3310	*R*	289.51
2	Me/OH	CH_3_	/	1.035	1589	*S*	1.045	1554	*R*	1.029	1650	*R*	266.08
3	Me/OMe	CH_3_	CH_3_	1.011	1578	*S*	1.021	1547	*R*	<1.01	1348	*R*	242.20
4	Me/Ac	CH_3_	OCH_2_CH_3_	1.021	1709	*S*	1.010	1630	*S*	1.015	1814	*S*	255.74
5	Me/TFA	CH_3_	OCH_2_CF_3_	1.016	1577	*S*	1.007	1409	*S*	1.01	1514	*S*	243.38
6	Et/OH	CH_2_CH_3_	/	1.036	1579	*S*	1.054	1552	*R*	1.025	1649	*R*	282.27
7	cyclic	1,3-dioxolane-2,4 dione		1.107	1709	*S*	1.041	1625	*S*	<1.01	1769		269.28

(a) The state of the carboxyl function; (b) the state of the hydroxyl function; (c) chiral selectivity value; (d) calculated boiling point (°C); (e) permethylated α-cyclodextrin-containing selector (α-CD); (f) permethylated β-cyclodextrin-containing selector (β-CD); (g) permethylated γ-cyclodextrin-containing selector (γ-CD). The identification numbers and abbreviations are the same as in Figure 1.

**Table 2:** Chiral selectivity, Kovats retention index values, and calculated boiling points, where mandelic acid methyl ester was substituted in its alkyl chain at 100 °C on various CD selectors.

ID:	Abrev.	α-CD ^d^		β-CD ^e^		γ-CD ^f^		bp ^c^
		Selectivity ^a^	KI ^b^	Selectivity ^a^	KI ^b^	Selectivity ^a^	KI ^b^	
2	Me/OH	1.035	1589	1.045	1554	1.029	1650	266.08
8	+Me/Me/OH	<1.01	1717	1.015	1623	<1.01	1754	282.27
9	tMe/Me/OH	1.048	1557	1.047	1522	1.012	1604	268.34

(a) Chiral selectivity value; (b) Kovats retention index; (c) calculated boiling point; (d) permethylated α-cyclodextin-containing selector (α-CD); (e) permethylated β-cyclodextin-containing selector (β-CD); (f) permethylated γ-cyclodextin-containing selector (γ-CD). The identification numbers and abbreviations are in Figure 1 and Figure 5.

**Table 3:** Chiral selectivity, Kovats retention indexes, elution orders, and their boiling points of ring-substituted mandelic acid methyl ester derivatives at 100 °C on various cyclodextrin selectors.

ID	Abr.	α-CD ^d^			β-CD ^e^			γ-CD ^f^			bp ^c^
		Selectivity ^a^	KI ^b^	Elution Order	Selectivity ^a^	KI ^b^	Elution Order	Selectivity ^a^	KI ^b^	Elution Order	
2	Me/OH	1.035	1589	S	1.045	1554	R	1.029	1650	R	266.08
10	2Cl/Me/OH	<1.01	1740		<1.01	1727		1.028	1803	R	291.72
11	3Cl/Me/OH	1.027	1829	S	1.141	1745	S	1.015	1741	R	291.72
12	4Cl/Me/OH	1.024	1807	S	1.170	1725	R	1.067	1866	R	291.72
13	3,4-diox/Me/OH	1023	1813		1.015	1949		1.007	1878		269.28

(a) Chiral selectivity value; (b) Kovats retention index; (c) calculated boiling point; (d) permethylated α-cyclodextin-containing selector (α-CD); (e) permethylated β-cyclodextin-containing selector (β-CD); (f) permethylated γ-cyclodextin-containing selector (γ-CD). The system of symbols is similar to Table 1 with prefixes. The first numbers show the positions of substitutions, which follow the chemical nature of the substitutions: Cl, chlorine; 3,4 diox, 3,4 dioxolane. The structures are in Figure 6.

## Data Availability

Data are contained within the article.
